# Quantitative assessment of preoperative brain development in pediatric congenital heart disease patients by synthetic MRI

**DOI:** 10.1186/s13244-024-01746-0

**Published:** 2024-07-02

**Authors:** Shengfang Xu, Zihan Ma, Jinlong Zhang, Shaoyu Wang, Xin Ge, Songhong Yue, Xinyi Li, Jifang Qian, Dalin Zhu, Guangyao Liu, Jing Zhang

**Affiliations:** 1 Second Clinical School, Lanzhou University, Lanzhou, Gansu China; 2 Department of Magnetic Resonance, Lanzhou University Second Hospital, Lanzhou, Gansu China; 3 Gansu Province Clinical Research Center for Functional and Molecular Imaging, Lanzhou, Gansu China; 4 Medical Imaging Center, Gansu Provincial Maternity and Child-Care Hospital, Lanzhou, Gansu China; 5 Pulmonary and Critical Care Medicine, The 940th Hospital of the Joint Logistic Support Force of the People’s Liberation Army, Lanzhou, Gansu China; 6 MR Research Collaboration, Siemens Healthineers, Shanghai, China

**Keywords:** Congenital heart disease, Infants, Brain development, Synthetic magnetic resonance imaging

## Abstract

**Objectives:**

This study investigated the quantitative assessment and application of Synthetic MRI (SyMRI) for preoperative brain development in children with congenital heart disease (CHD).

**Methods:**

Forty-three CHD patients aged 2–24 months were prospectively included in the observation group, and 43 healthy infants were included in the control group. The SyMRI scans were processed by postprocessing software to obtain T1, T2, and PD maps. The values of T1, T2, and PD in different brain regions were compared with the scores of the five ability areas of the Gesell Development Scale by Pearson correlation analysis.

**Results:**

In the observation group, the T1 values of the posterior limb of the internal capsule (PLIC), Optic radiation (PTR), cerebral peduncle, centrum semiovale, occipital white matter, temporal white matter, and dentate nucleus were greater than those in the control group. In the observation group, the T2 values of the PLIC, PTR, frontal white matter, occipital white matter, temporal white matter, and dentate nucleus were greater than those in the control group. Pearson correlation analysis revealed that the observation group had significantly lower Development Scale scores. In the observation group, the T2 value of the splenium of the corpus callosum was significantly positively correlated with the personal social behavior score. The AUCs for diagnosing preoperative brain developmental abnormalities in children with CHD using T1 values of the temporal white matter and dentate nucleus were both greater than 0.60.

**Conclusions:**

Quantitative assessment using SyMRI can aid in the early detection of preoperative brain development abnormalities in children with CHD.

**Critical relevance statement:**

T1 and T2 relaxation values from SyMRI can be considered as a quantitative imaging marker to detect abnormalities, allowing for early clinical evaluation and timely intervention, thereby reducing neurodevelopmental disorders in these children.

**Key Points:**

- T1 and T2 relaxation values by SyMRI are related to myelin development.
- Evaluated development quotient markers were lower in the observation compared to the control group.
- SyMRI can act as a reference indicator for brain development in CHD children.

**Graphical Abstract:**

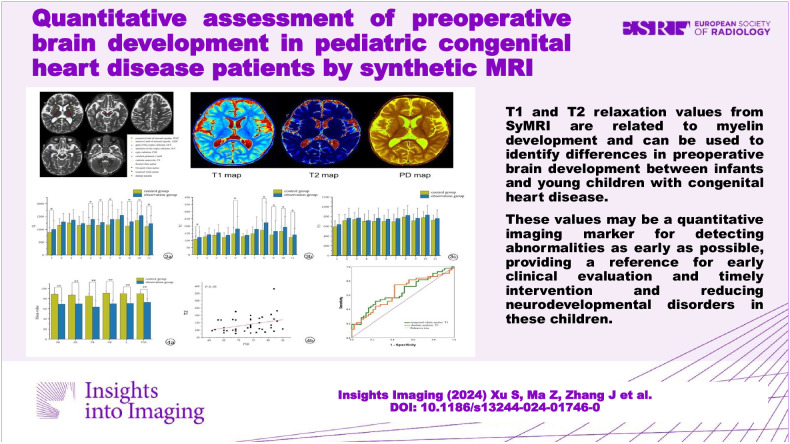

## Introduction

Congenital heart disease (CHD) is the most common congenital birth defect in infants and young children, and children with CHD can experience different degrees of neurodevelopmental disorders. Early detection and scientific evaluation are conducive to timely clinical intervention, which is crucial for improving neurological function, development, and the quality of life of children with CHD [1, 2]. The early clinical manifestations of nervous system dysplasia are not obvious, which complicates the diagnosis and treatment of this disease. Therefore, early assessment of the neurological development of children with CHD can aid in understanding the pathogenesis of neurological deficits and guide the early diagnosis of and timely intervention for neurological abnormalities [3–5].

Synthetic magnetic resonance imaging (SyMRI) is an emerging imaging modality that facilitates quantitative analysis of tissues by simultaneously obtaining multiple contrast images and multiple quantitative maps in a single scan over a short period. Studies on quantitative relaxation values in the central nervous system have been reported in the literature [6–8]. Quantitative information, such as longitudinal relaxation time (T1) and transverse relaxation time (T2) relaxation values, which can reflect changes in the myelin content of the brain, can aid imaging physicians in the detection of subtle brain lesions. Decreases in brain tissue moisture and myelin formation are the most important changes in infant brain development. T1 and T2 relaxation times reflect microstructural changes in water content, zoning, and macromolecules associated with early brain maturation and myelination [9]. Studies have shown that SyMRI can provide more quantitative indicators for the assessment of premature brain development, and these parameters can be used to plot brain development curves or identify brain diseases. A study by Lee et al provided T1, T2, and proton density (PD) reference values of brain regions from newborns to adolescents; these values are objective tools for assessing normal/abnormal brain development using SyMRI [10–13]. To date, there have been few studies on SyMRI in the assessment of brain development in infants with CHD. Through the quantitative evaluation and correlation analysis of the T1, T2, and PD values of SyMRI in the preoperative brain development of children with CHD, this study explored the feasibility and application value of synthetic MRI technology in assessing the preoperative brain development of infants with CHD.

## Materials and methods

### General information

A total of 43 children with CHD admitted to our hospital between February and October 2023 were prospectively enrolled in the observation group, and 43 healthy infants composed the control group. The inclusion criteria for the CHD group were as follows: ① age 2–24 months; ② confirmation of CHD by routine echocardiography; ③ head MRI scan quality meeting the diagnostic requirements; and ④ assessment of Gesell Development Schedules (Gesell) scores before operation. The inclusion criteria for the normal control group were as follows: ① age 2–24 months; ② no abnormalities in the cranial MR imaging results and quality of the MRI scan images meeting the diagnostic requirements; ③ normal Gesell scores; and ④ preterm infants with hypoxic-ischemic encephalopathy, hyperbilirubinemia, CHD, congenital craniocerebral malformation, congenital infectious diseases, chromosomal diseases, or congenital genetic metabolic diseases. This study used the Gesell to measure the Gesell development quotient (DQ) of the subjects in both groups [14]. Approval for this study was obtained from our institutional ethics committee, and informed consent was obtained from the parents or guardians.

### Magnetic resonance scan

MRI image acquisition: all subjects underwent routine MRI and SyMRI examinations on a 3.0-T MRI scanner (MAGNETOM Lumina, Siemens Healthcare). SyMRI utilized a multidynamic multiecho (MDME) sequence with the following scanning parameters: TR 4450 ms, TE 23 ms, receiver bandwidth 150 Hz/pixel, the field of view = 180 × 180 mm^2^, matrix size = 256 × 180, 30 slices, 3 mm slice thickness, 0.6 mm slice gap, and a total scan time of 4 min 50 s. To ensure the smooth completion of the examination and reduce the artifacts of head movement, infants and young children were sedated with oral chloral hydrate or enemas (20 mg/kg) 15–30 min before the scan. The infant was swaddled and placed on the MRI scanning bed after falling asleep. During the examination, hearing protection and warmth were provided.

### Image postprocessing

**Fig. 1:**
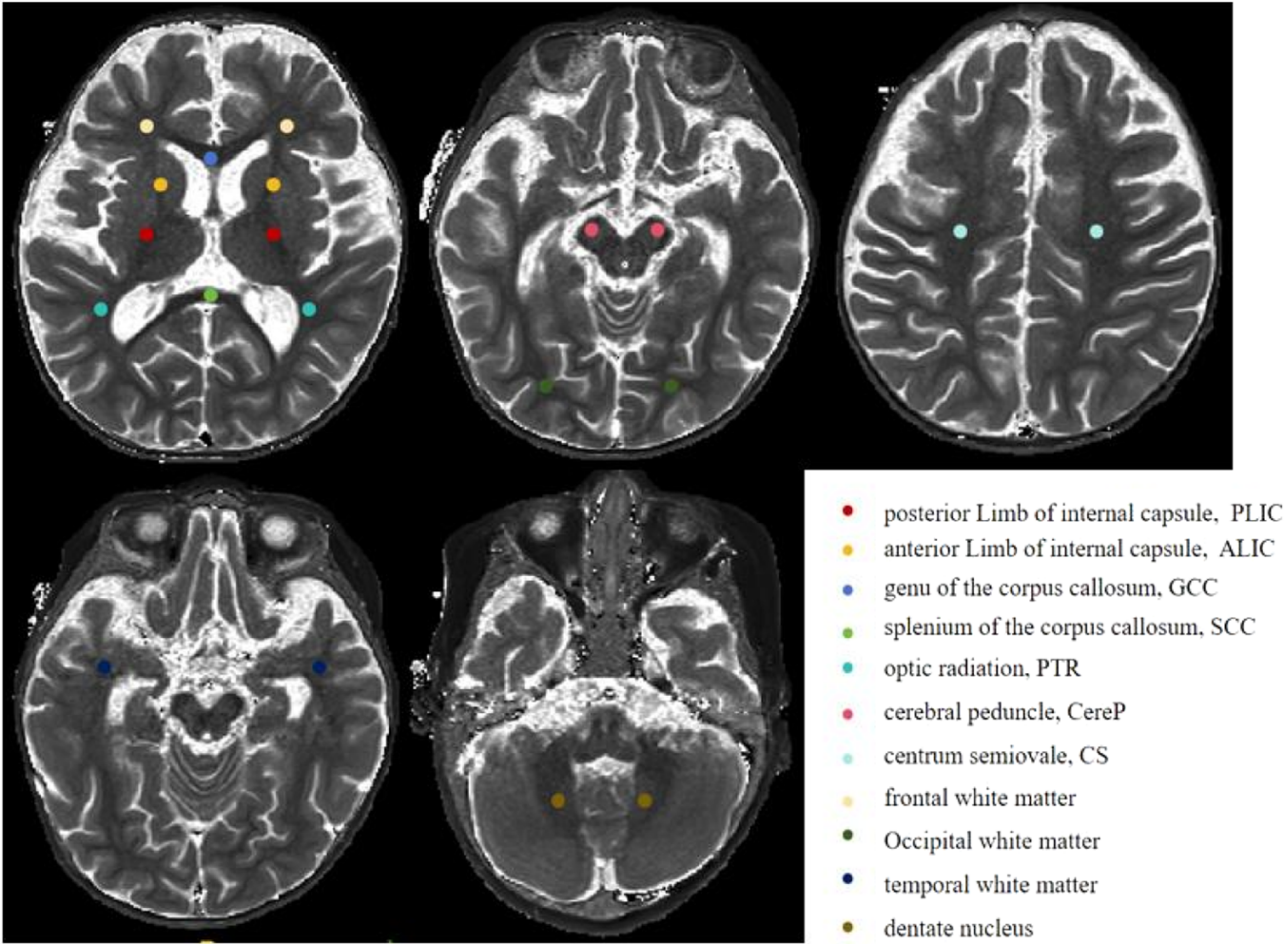
Schematic diagram of various brain region ROIs

**Fig. 2:**
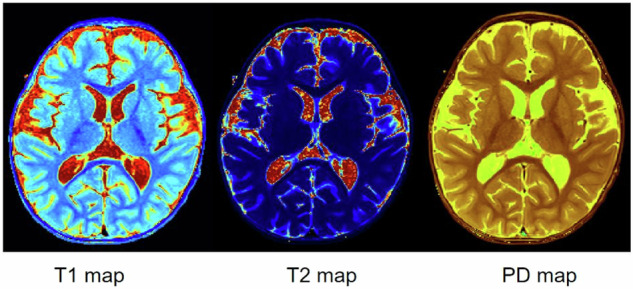
Pseudo-color maps of T1, T2, and PD by SyMRI

The acquired scan data were processed using an in-house MDME Toolbox (designed and provided by a collaboration scientist from Siemens Healthineers) to automatically calculate T1, T2, and PD maps. Manual delineation of two brain region of interests (ROIs) (Fig. 1) for both groups was performed on the parameter maps using ITK-SNAP 4.0.1 software, and the T1, T2, and PD values were computed, as shown in Fig. 2. ROIs were averaged at the anatomical centers of bilateral brain symmetry, except for the genu of the corpus callosum and splenium of the corpus callosum (SCC).

### Statistical analysis

SPSS 27.0 software was employed for data analysis. Continuous data are expressed as the mean ± standard deviation, while categorical data are presented as frequencies and percentages. Group differences in categorical data were assessed using chi-square analysis, and for continuous data, independent samples t-tests were conducted. Comparative analyses were performed on T1, T2, and PD values in different brain regions of interest (ROIs) and the five functional area scores from the Gesell between the two groups. Pearson correlation analysis was utilized to examine the relationship between the regions with differing T1, T2, and PD values and the scores in the five functional areas of the Gesell. Receiver operating characteristic (ROC) curves were generated to assess the diagnostic efficacy of T1 and T2 values in brain regions with statistically significant differences for identifying preoperative brain developmental abnormalities in children with CHD. A *p* value < 0.05 was considered to indicate statistical significance.

## Results

### General information

There were 21 males and 22 females in the observation group and 23 males and 20 females in the control group. The average age of the individuals in the observation group was 5.651 ± 3.664 months, and the average age of the individuals in the control group was 6.767 ± 3.872 months. There were no significant differences in age or sex between the two groups (*p* = 0.173 and *p* = 0.385). The parents of all enrolled children were healthy and had no comorbidities. One pair of twins in the CHD group had atrial septal defects.

In the observation group, there were 11 patients with atrial septal defect; 5 patients with ventricular septal defect; 4 patients with atrial septal defect and ventricular septal defect; 2 patients with patent ductus arteriosus; 1 patient with ventricular septal defect and patent ductus arteriosus; 5 patients with atrial septal defect and patent ductus arteriosus; 1 patient with atrial septal defect, ventricular septal defect, and patent ductus arteriosus; 1 patient with ventricular septal defect and tricuspid valve insufficiency; 1 patient with ventricular septal defect, atrial septal defect, and tricuspid valve insufficiency; 1 patient with ventricular septal defect, atrial septal defect, pulmonary atresia, and congenital collateral circulation of the pulmonary artery; 1 patient with ventricular septal defect and persistent left superior vena cava; 1 patient with patent ductus arteriosus, partial abnormality of the pulmonary vein junction, congenital bicuspid aortic malformation, and aortic stenosis; 1 patient with ventricular septal defect and congenital subaortic diaphragm; 1 patient with ventricular septal defect, congenital subaortic septum, and tricuspid valve insufficiency; 1 patient with ventricular septal defect, abnormal coronary artery origin, mitral valve insufficiency, and cardiac insufficiency; 1 patient with ventricular septal defect, atrial septal defect, patent ductus arteriosus, and mitral valve insufficiency; 1 patient with atrial septal defect, pulmonary stenosis, and patent ductus arteriosus; 1 patient with ventricular septal defect, atrial septal defect patent ductus arteriosus, and mitral valve insufficiency; 1 patient with congenital tricuspid valve atresia (Type Ia), ventricular septal defect, atrial septal defect, and right ventricular hypoplasia syndrome; 1 patient with aortic coarctation and ventricular septal defect; 1 patient with ventricular septal defect, atrial septal defect, and mitral valve insufficiency; and 1 patient with congenital aortic arch rupture, ventricular septal defect, atrial septal defect, unclosed ductus arteriosus, and tricuspid valve insufficiency.

The following abnormalities were also observed in the observation group: 1 case of cleft palate; 1 case of chromosomal abnormality and exotropia; 4 cases of congenital laryngeal chondromalacia; 4 cases of congenital bronchial hypoplasia and trachea dysplasia; 1 case of unilateral renal absence, congenital thoracic malformation, and congenital rib malformation; 1 case of intracranial hemorrhage; 1 case of cytotoxic edema of the corpus callosum, bilateral temporal-parietal-occipital encephalomalacia, and local cortical necrosis; and 1 case of Dandy–Walker syndrome and agenesis of the corpus callosum.

Magnetic resonance angiography revealed the following: 1 patient had a left embryonic posterior cerebral artery; 1 patient had a right embryonic posterior cerebral artery; 1 patient had a bilateral cerebral artery; 1 patient had a triploid anterior cerebral artery; 1 patient had a left transverse sinus that was not visible; 3 patients had a thin left transverse sinus; 1 patient had a straight sinus into the left transverse sinus; 1 patient had a straight sinus into the left sigmoid sinus; and 1 patient had a thin right intravenous vein, sigmoid sinus, and transverse sinus.

### Comparison of T1, T2, and PD values between the two groups

**Fig. 3:**
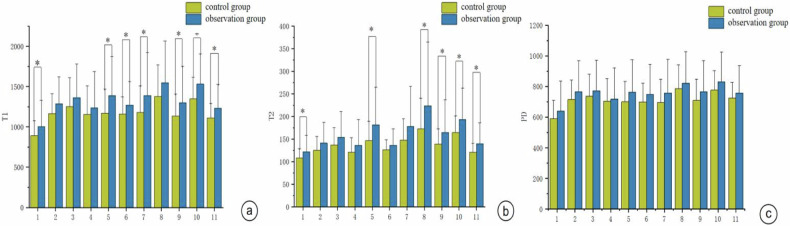
**a** Comparison of T1 values within various brain region ROIs in the two groups. **b** Comparison of T2 values within various brain region ROIs in the two groups. **c** Comparison of PD values within various brain region ROIs in the two groups (*Inter-group comparison *p < 0.05)

In the observation group, the T1 values of the posterior limb of the internal capsule (PLIC), optic radiation (PTR), cerebral peduncle (CereP), centrum semiovale (CS), occipital white matter, temporal white matter, and dentate nucleus were significantly greater than those in the control group (*p* < 0.05) (Fig. 3a). In the observation group, the T2 values of the PLIC, PTR, frontal white matter, occipital white matter, temporal white matter, and dentate nucleus were significantly greater than those in the control group (*p* < 0.05) (Fig. 3b).

### Correlation analysis

**Fig. 4:**
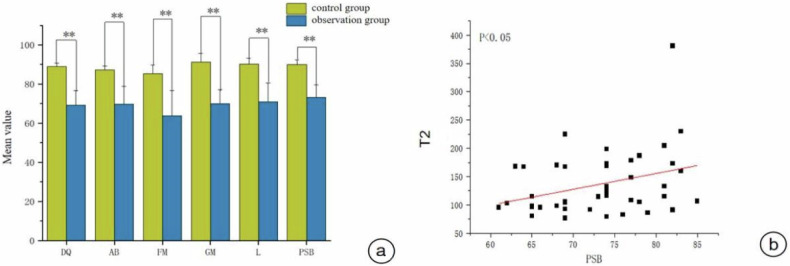
**a** Comparison of scores in various functional areas of the Gesell in the two groups (*inter-group comparison *p < 0.05, **p < 0.01). **b** Correlation analysis: positive correlation between T2 values in the SCC and PSB. DQ, development quotient; AB, adaptive behavior; FM, fine motor; GM, gross motor; L, language; PSB, personal-social behavior

Pearson correlation analysis revealed that the observation group had significantly lower scores for DQ, adaptive behavior (AB), fine motion (FM), great movement (GM), language (L), and personal social behavior (PSB) (*p* < 0.05) compared to those of the control group (Fig. 4). In the observation group, the T2 value of the SCC was significantly positively correlated with the PSB score (*r* = 0.443, *p* = 0.002) (Fig. 4).

### Diagnostic efficiency

**Fig. 5:**
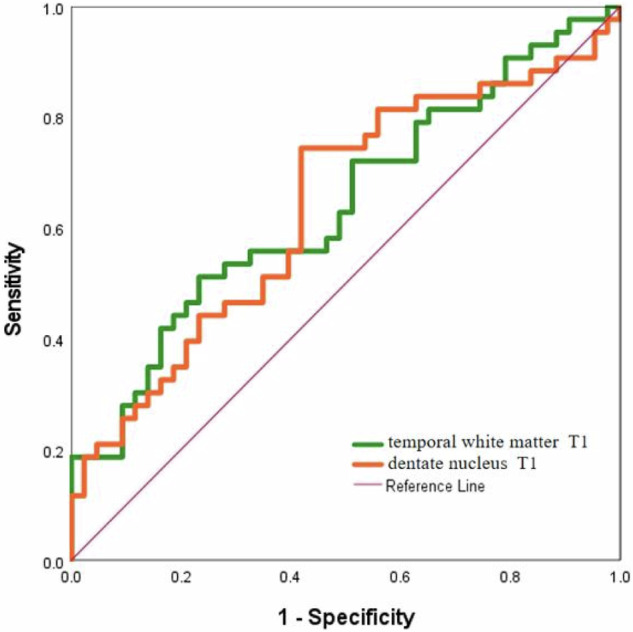
ROC curves for T1 and T2 values in various brain regions for diagnosing preoperative brain developmental abnormalities in children with CHD

The areas under the ROC curves (AUCs) for diagnosing preoperative brain developmental abnormalities in children with CHD using T1 values of the temporal white matter and dentate nucleus were both greater than 0.60 (Fig. 5).

## Discussion

Children’s brain development is characterized by myelination, neuronal maturation, and synapse formation. The myelin (MYE) sheath is a double-layered structure formed by the oligodendrocyte cell membrane in the central nervous system, which is embedded with several macromolecular proteins, and its outer layer is composed of glycolipids and cholesterol [15]. Decreased water content and myelination of brain tissue are the most important changes in infant brain development, and T1 and T2 relaxation times reflect changes in water content, partitioning, and macromolecule microstructure associated with early brain maturation and myelination [9, 16].

Therefore, myelin formation is an important step in the development of the central nervous system, and myelin is a key factor in assessing neural development. Since the maturation of MYE leads to a decrease in T1 and T2 relaxation times, water molecule diffusion and PD values, and an increase in diffusion anisotropy and magnetization transfer, MRI is a major tool for assessing myelin development [17, 18]. Lee et al studied SyMRI imaging of healthy neonatal to adolescent brains and found that, except for cortical PD values, T1, T2, and PD values decreased with age. They also provided age-specific reference values as an objective basis for assessing normal/abnormal brain development [13, 19]. Vanderhasselt et al reported that neonates with severe postnatal disorders had prolonged local tissue relaxation times, particularly in the PLIC and the central portion of the CS, which had the highest accuracy in predicting at-risk status [20]. Recent studies have shown that the T1 and T2 relaxation values obtained by SyMRI are closely related to the degree of myelin development, which can reflect subtle differences in early brain development and is an effective indicator for assessing brain maturity [8]. In this study, we quantitatively assessed the preoperative brain development of infants and children with CHD by SyMRI, compared the differences in relaxation values between the two groups of subjects in different brain regions, and analyzed the correlation between the significantly different parameter values and Gesell development scores. The T1 and T2 values of the posterior limb of the internal capsule, PTR, frontal white matter, occipital white matter, temporal white matter, and dentate nucleus of the cerebellum were greater in the observation group than in the control group, and the T1 values of the CereP and the center of the semiovale were greater in the observation group than in the control group. The relaxation values of T1 and T2 were prolonged in different parts of the brain in the two groups, suggesting the existence of subtle differences in brain structure between infants and children with preoperative CHD. Some studies have shown that the decrease of T1 and T2 relaxation values is related to myelin maturation and a reduction in water in the brain, and the effect of PD on brain maturation in neonates is less clear than that of T1 and T2 values. In the present study, PD did not significantly affect preoperative brain development in infants or children with CHD, and it is hypothesized that a decrease in PD may occur later or more slowly than a decrease in T1 or T2. Structural brain abnormalities in children with CHD have been associated with alterations in cerebral blood flow at critical moments of development and abnormalities in cerebral blood flow may lead to the inhibition of myelination [11, 21, 22]. The present study showed that some children with CHD had developmental abnormalities of the cerebral vasculature. The visual evaluation of myelin development based on image signal intensity in conventional MRI is imprecise, and the quantitative evaluation of myelin development using diffusion tensor imaging, magnetic resonance spectroscopy, and other imaging techniques requires a long scanning time. SyMRI can quantitatively analyze the T1, T2, and PD values of the whole brain in a single scanning session, which provides a new approach to the detection and monitoring of myelin developmental abnormalities [23].

The early clinical manifestations of nervous system dysplasia are not obvious, which complicates the diagnosis and treatment of this disease. Therefore, the scientific evaluation of the neurodevelopmental status of CHD children before surgery may assist in guiding the early diagnosis and timely intervention of neurodevelopmental abnormalities. At present, there are numerous developmental scales for evaluating the development of the nervous system in infants and young children. In this study, the Gesell scale was used for evaluation. Through the quantitative evaluation and correlation analysis of the T1, T2, and PD values of SyMRI in the preoperative brain development of children with CHD, the correlation between each parameter and the Gesell development quotient (DQ) was determined to predict early nervous system developmental abnormalities in children with CHD. The DQ, AB, FM, GM, L, and PSB scores of the observation group were lower than those of the control group. The increase in T2 relaxation value in the corpus callosum pressure area of the observation group was significantly positively correlated with PSB scores, indicating the presence of neurodevelopmental delay in infants and young children with CHD before surgery. The ROC curve showed that the T1 values of the temporal lobe white matter and cerebellar dentate nucleus had good diagnostic efficacy for preoperative brain developmental abnormalities in children with CHD.

This study has several limitations: the sample size was small, the types of CHD in infants and young children were not classified, there are many factors that can affect relaxation time, the follow-up time for infants and young children was relatively short, and the long-term neurological development status of the children was not observed during follow-up. Therefore, it is necessary to increase the sample size for longitudinal long-term neurological development follow-up in the future. In future studies, we will expand the sample size to improve the reliability and collect SyMRI quantitative data to support the application of deep learning in the preoperative assessment of brain development in infants and toddlers with CHD. In addition, multimodal parameters will be combined with other technical methods (such as diffusion kurtosis imaging and neurite direction dispersion and density imaging) and compared to refine the preoperative brain development of infants and toddlers with CHD.

## Conclusion

In summary, the T1 and T2 relaxation values obtained by integrated MRI technology are related to myelin development and can be used to identify subtle differences in preoperative brain development between infants and young children with CHD. These values may be a quantitative imaging marker for detecting abnormalities as early as possible, providing a reference for early clinical evaluation and timely intervention thereby reducing neurodevelopmental disorders in these children.

## Data Availability

The datasets generated or analyzed during the study are available from the corresponding author upon reasonable request.

## Abbreviations

AB: Adaptive behavior
CereP: Cerebral peduncle
CHD: Congenital heart disease
CS: Centrum semiovale
DQ: Development quotient
FM: Fine motion
GM: Great movement
L: Language
MDME: Multi-dynamic multi-echo
MRI: Magnetic resonance imaging
PD: Proton density
PLIC: Posterior limb of internal capsule
PSB: Personal social behavior
PTR: Optic radiation
ROI: Region of interest
SCC: Splenium of the corpus callosum
SyMRI: Synthetic magnetic resonance imaging
T1: Longitudinal relaxation time
T2: Transverse relaxation time
